# Role of Platelet-Rich Plasma in the Treatment of Adhesive Capsulitis: A Prospective Cohort Study

**DOI:** 10.7759/cureus.30542

**Published:** 2022-10-21

**Authors:** Syed Imran Haider, Muhammad Zarak Awais, Muhammad Tahir Iqbal

**Affiliations:** 1 Department of Orthopedic Surgery, Mayo Hospital, Lahore, PAK

**Keywords:** platelet rich plasma, pain, glenohumeral, frozen shoulder, adhesive capsulitis

## Abstract

Background

A glenohumeral (GH) joint disease, such as adhesive capsulitis, causes the shoulder capsule's fibrosis and adhesion to restrict mobility. Collagen and growth factors can be produced by platelet-rich plasma (PRP), which promotes stem cells and, as a result, improves the healing process. This study was done to determine the role of PRP injection in the treatment of adhesive capsulitis in terms of improvement in pain.

Methodology

This prospective cohort study was conducted at the Department of Orthopedics, Mayo Hospital, Lahore, Pakistan, from February 2022 to July 2022. A total of 305 patients were enrolled through the outpatient department. Basic demographic and clinical details, including the visual analog scale (VAS) pain score, were noted. First, 20 ml of the patient’s blood was drawn from the superficial saphenous vein by double syringe. After processing, the PRP was collected and injected in the subacromial bursa and intra-articular space adopting the anatomical approach. Then, the process was repeated at weekly intervals for four weeks. In this phase, PRP was injected only in the joint. The pain was assessed before and after six weeks of treatment and improvement in pain as per VAS was noted.

Results

In a total of 305 cases, the mean age was 60.47±11.55 years. There were 164 (53.8%) male and 141 (46.2%) female cases. The mean pain VAS scores before and after treatment were 6.56±1.79 and 2.42±1.71, respectively, and the mean reduction in pain after treatment was 64.57±19.40%. In 267 (87.5%) cases, an improvement of ≥ 50% was observed while 38 (12.5%) cases had an improvement of <50%.

Conclusion

The PRP can be used for the treatment of adhesive capsulitis to reduce pain. This non-operative method of treatment may help reduce the hospital burden of patients suffering from adhesive capsulitis.

## Introduction

Adhesive capsulitis is also known as frozen shoulder and is described as a disorder of the glenohumeral joint, which restricts movement due to adhesion and the presence of fibrosis in the shoulder capsule [1,2]. Manual therapy and exercise are commonly carried out to treat adhesive capsulitis [3,4]. It is categorized as primary (idiopathic) or secondary; the latter is caused by rotator cuff tears, hemiparesis, cardiovascular diseases, and diabetes mellitus [5]. Among the general public, the occurrence of adhesive capsulitis has been found to be 2-5% with a dominance of the female factor as compared to the male [1].

There are different approaches to treating adhesive capsulitis; the primary objective is to provide relief from pain and to make the joint functional whether it is non-operative or an operative intervention [6]. Many cases of adhesive capsulitis are treated with conservative treatment using different therapeutic injections in the joint or physiotherapy [7]. Recent data have reported that PRP can help in producing collagen and growth factors, capable of increasing the capacity of stem cells to speed up the healing process [2]. A recent case report reported that with the administration of the first dose of the injection, a 60% reduction of the diurnal shoulder pain was reported by the patient [2]. As data are lacking, only a case report is in view, so we conducted a pilot study in which 15 cases were taken [2]. In the sixth week, a reduction in pain of ≥ 50% was noted in 11 (73.3%) cases.

The rationale of our study was to see the role of PRP in our local population, as data are not available regarding the use of PRP in adhesive capsulitis. PRP is widely used in many orthopedically diseased populations, so this non-operative method of treatment may help reduce the hospital burden and can minimize the risk of limb disability. For the patients, this may contribute to an improvement in their quality of life. This study was done to define the benefits of injectable PRP in order to treat adhesive capsulitis and associated pain.

## Materials and methods

This prospective cohort study was conducted at the Department of Orthopedics, Unit-1, Mayo Hospital, Lahore, Pakistan, from February 2022 to July 2022. The Mayo Hospital, Lahore, is affiliated with King Edward Medical University (KEMU), Lahore. Approval from the institutional review board was acquired (letter number: KEMU/2021/RC/328). Informed and written consent was sought from all study participants. The non-probability consecutive sampling technique was adopted. A sample size of 300 cases was taken considering the improvement in pain as 73.3% (as per the findings of the pilot study) with a 95% confidence level and a 5% margin of error. We enrolled 305 cases for this study adopting the non-probability consecutive sampling technique.

Inclusion criteria were patients of either gender aged 40-80 years having a frozen shoulder with a duration of six weeks or more. Exclusion criteria were pregnant or lactating females (as per history). Patients with a history of previous treatment for a frozen shoulder (on medical record) or systemic inflammation and/or osteoarthritis in other joints (assessed on the patient’s medical records) were also excluded. Patients losing follow-ups and not completing the full treatment duration were also excluded.

Patients were enrolled from the outpatient department and their basic demographic and clinical details (pain and assessment for frozen shoulder) were noted. Adhesive capsulitis was defined if there was pain > 3 on the visual analog scale (VAS) in the shoulder joint and there was a restricted passive and active range of motion. First, 20 ml of the patient’s blood was drawn from the superficial saphenous vein by double syringe. Next, the blood sample was centrifuged at 5000 rounds per minute (rpm) for five minutes to separate the blood into layers of red blood cells, buffy-coat of leucocytes, and plasma and lastly PRP (leucocyte rich) was collected and injected this product in the subacromial bursa and intra-articular space adopting an anatomical approach. Then, the process was repeated weekly for four weeks. In this phase, PRP was injected only in the GH joint. Shoulder stretching exercise was recommended to the patient after every injection. The pain was assessed before and after six weeks of treatment and improvement was labeled. The outcome was determined in terms of improvement in pain and measured on VAS (0-10, where 0 shows no pain, and 10 shows the worst pain) and improvement was labeled if there was a ≥ 50% reduction in the pain after treatment at the sixth week. All data were collected on a predesigned proforma.

All collected data were entered and analyzed in Statistical Package for Social Sciences (SPSS) version 26.0 (IBM Corp., Armonk, NY). Mean ± standard deviation (SD) was used for quantitative data such as age and pain (before and at the sixth week). Frequency (%) was calculated for qualitative data like gender and improvement in pain (≥ 50%) pain at the sixth week. Data were stratified for age, gender, duration of disease, and baseline pain to address effect modifiers. The chi-square test after stratification was applied to compare the improvement in pain after the treatment, taking p-value ≤0.05 as significant. The paired sample t-test was applied to compare baseline and after-treatment VAS pain scores considering p-value≤0.05 as statistically significant.

## Results

In a total of 305 cases, the mean age was 60.47±11.55 years (ranging between 40 and 80 years). There were 164 (53.8%) male patients while the male-to-female ratio was calculated to be 1.16:1. Table 1 shows the baseline characteristics of patients.

**Table 1:** Baseline Characteristics of Patients (n=305)

Baseline Characteristics		Number (%)
Age groups (years)	40-65	194 (63.6%)
	66-80	111 (36.4%)
Gender	Male	164 (53.8%)
	Female	141 (46.2%)
Duration of Disease	<12 weeks	170 (55.7%)
	≥12 weeks	135 (44.3%)
Pain Score (Visual Analogue Scale)	<6	104 (34.1%)
	6-10	201 (65.9%)

The mean baseline pain score was 6.56±1.79 (ranging between 4 and 9) while after treatment at six weeks, it was 2.42±1.71 (ranging between 1 and 7), p<0.001. Overall, a reduction of 64.57±19.40% was noted in the VAS pain score before and after the treatment. In 267 (87.5%) cases, the improvement was ≥ 50% while 38 (12.5%) cases had improvement <50%.

When cases with a ≥ 50 improvement in pain were compared with those having <50% improvement in pain, no statistically significant differences were found with regards to the distribution of age (p=0.434), gender (p=0.880), duration of disease (p=0.092), and baseline pain scores (p=0.279). The details about the comparison of pain improvement with respect to study variables are shown in Table 2. None of the patients experienced side effects or complications like infection, redness, or swelling.

**Table 2:** Comparison of pain improvement ≥ 50% with respect to age groups, gender, duration of disease, and baseline pain VAS score (N=305) VAS: visual analog scale

Study Variables		Pain Improvement ≥50%		P-Value
		Yes (n=267)	No (n=38)	
Age Groups (years)	40-65	172 (64.4%)	22 (57.9%)	0.434
	66-80	95 (35.6%)	16 (42.1%)	
Gender	Male	144 (53.9%)	20 (52.6%)	0.880
	Female	123 (46.1%)	18 (47.4%)	
Duration of Disease	<12 weeks	144 (53.9%)	26 (68.4%)	0.092
	≥12 weeks	123 (46.1%)	12 (31.6%)	
Baseline VAS Pain Score	<6	94 (35.2%)	10 (26.3%)	0.279
	6-10	173 (64.8%)	28 (73.7%)	

## Discussion

The conventional approach in the treatment of frozen shoulders is to provide relief from pain and to reinstate the movement of the joint for its proper functioning [8-10]. Multiple techniques like exercise, electrical healing, and massage are used from the very beginning if the patient with a frozen shoulder is being treated through physiotherapy. With the help of massages, heat therapy, cryotherapy, ultrasonic rays, transcutaneous electrical nerve stimulation (TENS), and light amplification by stimulated emission of radiations as mentioned in the textbooks or other concerned literature, only the associated pain might be reduced [11,12]. This perhaps is a short benefit to have [13]. Most of the time, these methods are further linked to manual therapeutic practices or exercise programs at home [13]. Physiological improvement of the shoulder joint is achieved by the introduction of ultrasonic rays, which increase the flow of blood, enhance the permeability of capillaries, improve tissue-based metabolic functions, make the tissue more flexible, bring a rise in the pain threshold, and modify neuromuscular activity, which causes the muscles to relax [14]. To treat adhesive capsulitis, one of the most frequently used methods is the administration of intra-articular corticosteroid injection [15]. PRP has grown to be the latest technique to treat frozen shoulders; it is believed that it stimulates the revascularization of soft tissue and enhances the growth factors' concentration, which provides an improvement in the speedy healing of tendons. Basically, PRP is a sample of an individual’s own blood with a higher concentration of platelets in comparison with normal blood [16].

A recent case report reported that after the first injection, the patient reported a 60% improvement in diurnal shoulder pain [2]. As data are lacking, with only a case report published, we conducted a pilot study in which 15 cases were considered. In the sixth week, a reduction in pain of ≥50% was noted in 11 (73.3%) cases. The mean age of our study cases was 60.47±11.55 years with minimum and maximum ages of 40 and 80. There were 164 (53.8%) male and 141 (46.2%) female cases. In 267 (87.5%) cases, the improvement in pain was ≥ 50% while 38 (12.5%) cases had pain improvement <50%. The results are better than those found in our pilot study.

In 2017, a study was conducted involving 54 female patients with frozen shoulders to evaluate the effectiveness of PRP [17]. Three PRP injections were administered at a seven-day interval while the Quick Dash questionnaire was utilized to record the pre-intervention and post-intervention data after 30 days, three months, and 12 months following treatment. Post-intervention Quick Dash scores were significantly improved following PRP treatment [17]. The authors came to the conclusion that the use of PRP was effective in reducing pain as well as functional outcomes among women with frozen shoulders. Potentially, adding PRP therapy with other therapeutic choices like analgesics and/or physical therapy may lead to improved outcomes among patients with frozen shoulders. As we noted relatively short-term outcomes in the present set of patients with frozen shoulders adopting PRP therapy, the findings of Jakovljevic A et al., employing 12-month follow-up data further strengthen what we found in this study [17].

A study by Kothari SY et al., comparing the effectiveness of PRP injection (2 ml single injection), corticosteroids (80 mg methylprednisolone), and ultrasonic therapy (7 sittings in 2 weeks at 1.5 W/cm^2^, 1 MHz, continuous mode) for the treatment of periarthritis shoulder were randomly divided into study groups [18]. Outcome measures were labeled at baseline and three-week, six-week, and 12-weeks intervals. The PRP treatment group had significantly better functional outcomes and VAS pain scoring in comparison to the other two study groups during the follow-up periods. The researchers concluded that single PRP injection therapy was more beneficial when compared to corticosteroid injection or ultrasonic treatment among patients with periarthritis shoulder [18].

Limitations

As this was a single-center study with a relatively small follow-up period, our findings cannot be generalized and studies involving multiple centers and longer duration of follow-ups should be planned to further verify the findings of this study.

## Conclusions

It is concluded that PRP can be used for the treatment of adhesive capsulitis to reduce pain. The PRP being a nonoperative method of treatment may help reduce the hospital burden of patients suffering from adhesive capsulitis. This can further help improve the quality of life of adhesive capsulitis patients. There is a need for exploring the further pathophysiological impact of PRP treatment in large multicentral cohorts to examine the best approach for the most improved outcomes.
